# QSAR Evaluations to Unravel the Structural Features in Lysine-Specific Histone Demethylase 1A Inhibitors for Novel Anticancer Lead Development Supported by Molecular Docking, MD Simulation and MMGBSA

**DOI:** 10.3390/molecules27154758

**Published:** 2022-07-25

**Authors:** Rahul D. Jawarkar, Ravindra L. Bakal, Nobendu Mukherjee, Arabinda Ghosh, Magdi E. A. Zaki, Sami A. AL-Hussain, Aamal A. Al-Mutairi, Abdul Samad, Ajaykumar Gandhi, Vijay H. Masand

**Affiliations:** 1 Department of Medicinal Chemistry and Drug Discovery, Dr Rajendra Gode Institute of Pharmacy, University Mardi Road, Amravati 444603, India; rlbakal@gmail.com; 2 Department of Microbiology, Ramakrishna Mission Vivekananda Centenary College, Kolkata 700118, India; nabendu21@rkmvccrahara.org; 3 Department of Health Sciences, Novel Global Community Educational Foundation, Hebersham, NSW 2770, Australia; 4 Microbiology Division, Department of Botany, Gauhati University, Guwahati 781014, India; dra.ghosh@gauhati.ac.in; 5 Department of Chemistry, Faculty of Science, Imam Mohammad Ibn Saud Islamic University, Riyadh 13318, Saudi Arabia; sahussain@imamu.edu.sa (S.A.A.-H.); aamutairi@imamu.edu.sa (A.A.A.-M.); 6 Department of Pharmaceutical Chemistry, Faculty of Pharmacy, Tishk International University, Erbil 44001, Iraq; abdul.samad@tiu.edu.iq; 7 Department of Chemistry, Government Arts and Science College, Karur 639005, India; gacjay18@gmail.com; 8 Department of Chemistry, Vidyabharati Mahavidyalalya, Camp, Amravati 444602, India; vijaymasand@gmail.com

**Keywords:** LSD1, KDM1A, QSAR, anticancer, molecular docking, MD simulation, genetic algorithm–multi linear regression, MMGBSA

## Abstract

Using 84 structurally diverse and experimentally validated LSD1/KDM1A inhibitors, quantitative structure–activity relationship (QSAR) models were built by OECD requirements. In the QSAR analysis, certainly significant and understated pharmacophoric features were identified as critical for LSD1 inhibition, such as a ring Carbon atom with exactly six bonds from a Nitrogen atom, partial charges of lipophilic atoms within eight bonds from a ring Sulphur atom, a non-ring Oxygen atom exactly nine bonds from the amide Nitrogen, etc. The genetic algorithm–multi-linear regression (GA-MLR) and double cross-validation criteria were used to create robust QSAR models with high predictability. In this study, two QSAR models were developed, with fitting parameters like R^2^ = 0.83–0.81, F = 61.22–67.96, internal validation parameters such as Q^2^_LOO_ = 0.79–0.77, Q^2^_LMO_ = 0.78–0.76, CCC_cv_ = 0.89–0.88, and external validation parameters such as, R2ext = 0.82 and CCCex = 0.90. In terms of mechanistic interpretation and statistical analysis, both QSAR models are well-balanced. Furthermore, utilizing the pharmacophoric features revealed by QSAR modelling, molecular docking experiments corroborated with the most active compound’s binding to the LSD1 receptor. The docking results are then refined using Molecular dynamic simulation and MMGBSA analysis. As a consequence, the findings of the study can be used to produce LSD1/KDM1A inhibitors as anticancer leads.

## 1. Introduction

Lysine-specific histone demethylase 1A (LSD1), also known as lysine (K)-specific demethylase 1A (KDM1A), is a crucial member of the monoamine oxidases family. LSD1 catalyzes two important and completely opposing enzymatic reactions with flavin adenine dinucleotide (FDA) as a cofactor: transcription repression via de-methylation at histone 3 lysine 4 methyl 1/2 (H3K4me1/2) and transcription activation via de-methylation at histone 3 lysine 9 methyl 1/2 (H3K9me1/2) [1]. LSD1 is also involved in the de-methylation of TP53, E2F1, and DNMT1 [2]. The typical healthy physiological condition is characterized by regimented epigenetic control of cyclic cellular processes, including rejuvenation, differentiation, and proliferation. LSD1 modulates differentiation and proliferation pathways in highly proliferative and widely metastatic small-cell lung cancer (SCLC) [3]. LSD1 overexpression is also found in other cancer types, including prostate cancer, breast cancer, colorectal cancer, and neuroblastoma [1,2,3].

According to reports, if LSD1 overexpression is reduced in several forms of blood malignancies (haematological sarcomas) such as leukaemia, multiple myeloma, and solid tumours, cell differentiation is also reduced. This makes LSD1 an appealing target for anticancer medication development [1].

There have been several reports of reversible LSD1 inhibitors to date [4]. Sorna et al. used high-throughput virtual screening to identify reversible LSD1 inhibitors; however, these drugs appeared to have considerable off-target and nonspecific effects [4,5]. Ma et al. found several pyrimidine–thiourea hybrids that showed a high sensitivity to LSD1 inhibition in vitro and in tumour xenografts. [4]. Furthermore, Li et al. developed a series of [1,2,3] triazolo [4,5-d] pyrimidine derivatives as selective LSD1 inhibitors, which were reported to block tumour cell migration [5]. The activity of documented reversible inhibitors, on the other hand, did not meet the advances of covalent inhibitors, due in part to the huge size and polarity of the LSD1 substrate binding pocket [6]. Furthermore, the reported compounds’ erroneous binding techniques and the lack of theoretical research motivated us to hunt for hidden and buried structural features that are required for developing effective, efficient, and reversible LSD1 inhibitors. This convergence of circumstances encouraged us to do a computational study on the proven reversible LSD1 inhibitors from the appropriate database, as well as examine the underlying structural elements influencing the design and inhibition of potent and effective LSD1 inhibitors.

QSAR is a statistically-based intersectional plan of activities and standardized technique for identifying the mathematical relationship between the structural property of a molecule and its biological activity. General QSAR modelling protocol involves: (I) selecting a sufficiently abundant, admissible molecular dataset with accurate biological activity; (II) 3D-structure creation and optimization; (III) molecular descriptor calculation and constrained trimming using appropriate statistical methods; (IV) QSAR model development using an algorithm that fits favourable molecular descriptors; and (V) sufficient validation of the existing QSAR model (s) [5]. Circumstantial QSAR analysis quantifies the relationship between conspicuous but seemingly confusing molecule structural features and their experimentally studied biological activity. Statistical QSAR analysis predicts the biological activity of a drug prior to wet lab manufacturing and experimental in vivo testing. A QSAR that is conceptually neutral, illustrative, and statistically enhances pharmacokinetics knowledge [6,7]. This emphasizes the value of the QSAR study in promoting lead optimization.

**Figure 1:**
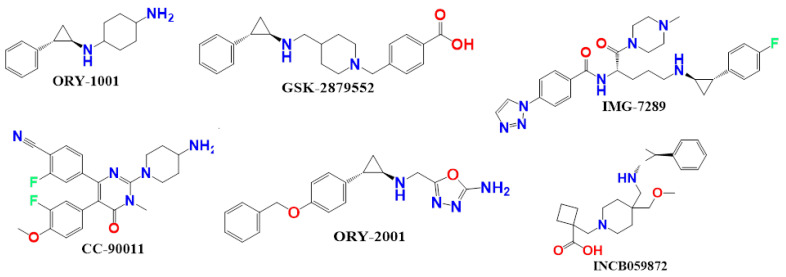
Presentation of the Structures of some clinical trial molecules.

QSAR models for LSD1 inhibitors have been discussed by a number of researchers. Rahman Abdizadeh et al. developed a 3D QSAR model for the set of tranylcypromine derivatives as a data set that performed similarly to the CoMFA (q^2^ = 0.67; r^2^ncv = 0.93; r^2^pred = 0.97), CoMFA-RF (q^2^ = 0.69; r^2^ncr = 0.93; r^2^pred = 0.93), CoMSIA (q^2^ = 0.83; r^2^ncv =0.96; r^2^pred = 0.96), and HQSAR models (q^2^ = 0.85; r^2^ncv,= 0.90; r^2^pred = 0.73) for training as well as the test set of LSD1 inhibition. Moreover, the significant gap between the q^2^ and the r^2^ values indicate the overfitting in both the COMFA and CoMSIA models. However, because of the absence of mechanistic interpretation and atom-by-atom pharmacophoric features in CoMFA and CoMSIA investigations, their use has been limited to the optimization of a few pharmacological classes [8]. To date, several LSD1 inhibitors have been approved, and some of them, including ORY-1001, GSK-2879552, IMG-7289, INCB059872, CC-90011, and ORY-2001 (See Figure 1), are currently being studied in clinical trials for cancer treatment, particularly in small lung cancer cells (SCLC) and acute myeloid leukaemia (AML) [9].

A moderate-sized dataset-based QSAR with enough predictive capability and mechanistic understanding is clearly useful for boosting lead potency. In this study, we used molecular docking, MD simulation, and MMGBSA to create robust QSAR models for 84 structurally varied molecules with empirically established LSD1 inhibitory efficacy.

## 2. Results

**Figure 2:**
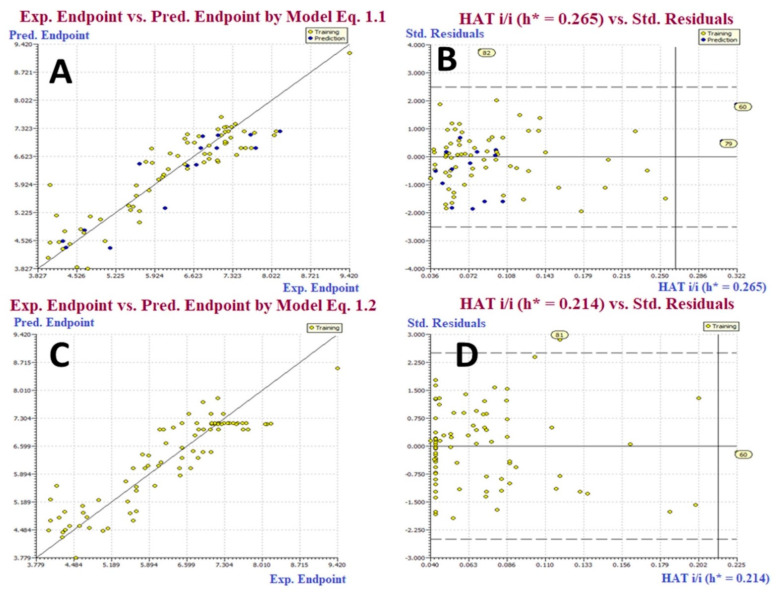
(**A**) Graph of experimental vs. Predicted pEC_50_ values for model 1.1. (**B**) Williams plot for model 1.1. (**C**) Graph of experimental vs. Predicted pEC_50_ values for model 1.2. (**D**) Williams plot for model 1.2.

Despite the fact that the current study is based on a moderate size dataset of 85 molecules, the existence of multiple molecular scaffolds, functional groups, substituents, diverse rings viz. non-aromatic, homoaromatic, heteroaromatic, fused rings; spiro compounds, etc., has significantly covered a vast chemical space. The QSAR models developed are based on a split and entire data set. R^2^, R^2^adj, CCC_tr_, and other fitting metrics have values far above the allowed threshold values, indicating that the QSAR models are statistically tolerable with the required number of chemical descriptors. Internal validation parameters include Q^2^_LOO_, Q^2^_LMO_, and others with values that condescend to give the statistical robustness of the QSAR models. The external predictability of both models can be seen in the high values of external validation aspects like R^2^_ex_ and Q^2^_Fn_. Model applicability domain is supported by William’s plots (See Figure 2) (Applicability Domain). Fulfillment of allowed threshold values for numerous parameters, as well as poor correlation among molecular descriptors, rule out the possibility of serendipitous QSAR model construction [10,11,12,13,14] (see Table S2, Supplementary Information). These grounds validate these models’ statistical robustness and strong external prediction.

### 2.1. Outlier Behavior of the Dataset Molecules

The third type of outlier, outliers toward the model, can only be identified after the regression model has been established. They indicate an X-Y link. Because of the variety of chemical structures explored in the study, model outliers are a specific form of outlier that may be found in high numbers in the QSAR/QSPR data set.

**Figure 3:**
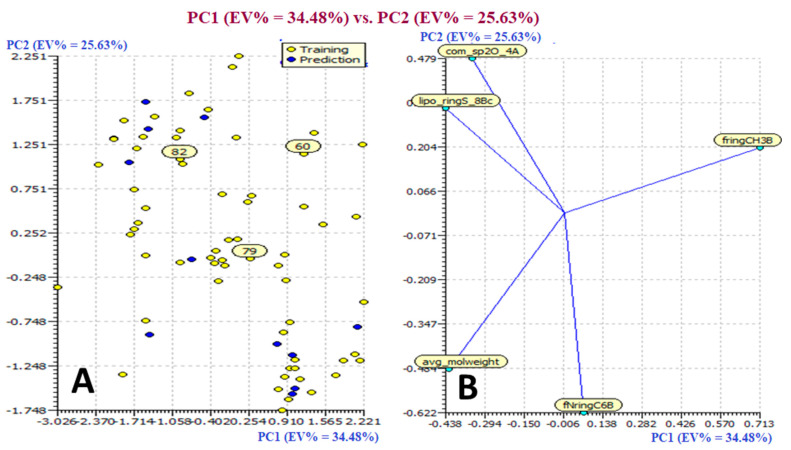
Presentation of Score Plot (**A**) and Loading Plot (**B**) for the Descriptor in divided set QSAR Model.

Based on the Williams plot, molecule 60 was identified as the third type of outlier in the divided set model, molecule 79 as an X outlier, and molecule 82 as a Y outlier. Figure 3 illustrates the core plot and loading plot of the Descriptor in a split-set QSAR model. The descriptor ring, CH3B, has a significant impact on molecule 60’s outlier characteristics, but the descriptors lipo_ringS_8Bc and com_sp2O_4A have a substantial impact on molecule 82. The descriptor **fringCH3B**, on the other hand, had a major impact on molecule 79. The aforementioned conclusion explained the impact of particular molecular descriptors on the cluster of molecules in the dataset (See Figure 3).

### 2.2. GA-MLR QSAR Models

Model-1.1 (Divided Set: Training Set-80% (67 molecules) and Prediction Set-20% (17 molecules)):

pEC_50_ = 13.856 (±1.734)–0.832 (±0.226) **avg_molweight**—0.211 (±0.087) **fringCH3B** + 0.263 (±0.092) **fNringC6B** + 4.482 (±1.892) **lipo_ringS_8Bc**—0.639 (±0.279) **com_sp2O_4A.**

[R^2^ = 0.83, R^2^_adj_ = 0.82, Q^2^_LOO_ = 0.79, Q^2^_LMO_ = 0.78, RMSE_tr_ = 0.49, MAE_tr_ = 0.37, RSS_tr_ = 16.37, CCC_tr_ = 0.91, RMSE_cv_ = 0.54, MAE_cv_ = 0.40, PRESS_cv_ = 20.07, CCC_cv_ = 0.89, R^2^_ext_ = 0.82, $Q_{F^{1}}^{2}=0.81$, $Q_{F^{2}}^{2}=0.81,\;Q_{F^{3}}^{2}=0.81$, CCC_ex_ = 0.90].

Model-1.2 (Full Set: Training Set-100%, (84 molecules)):

**pEC_50_** = 6.488 (±0.315)–0.151 (±0.078) **fringCH3B** + 2.921 (±1.496) **lipo_ringS_8Bc** + 0.972 (±0.349) **famdNnotringO9B** + 0.347 (±0.104) **fdonsp3C2B**—0.775 (±0.302) **fsp3CamdN4B.**

[R^2^ = 0.81, R^2^_adj_ = 0.80, Q^2^_LOO_ = 0.78, Q^2^_LMO_ = 0.77, RMSE_tr_ = 0.51, MAE_tr_ = 0.41, RSS_tr_ = 22.24, CCC_tr_ = 0.90, RMSE_cv_ = 0.56 MAE_cv_ = 0.45, PRESS_cv_ = 26.57, CCC_cv_ = 0.88].

In this QSAR investigation, model 1.1 was constructed using the extended dataset, whereas model 1.2 was created using the entire dataset. The developed models are distinct in three of the five descriptors out of a total of five. The effects of variation in each molecular descriptor on the biological activity of the associated molecule are demonstrated with examples in the next section, even if the permutation in the bioactivity of each molecule in the dataset is the total of all five molecular descriptors.

## 3. Discussion

### 3.1. Mechanistic Interpretation of Descriptors

**fNringC6B, lipo_ringS_8Bc, famdNnotringO9B, and fdonsp3C2B**: These four molecular descriptors had positive coefficient values in both the divided and full set models, showing that amplification in the values of these molecular descriptors improves the anticancer potential of LSD1 inhibitors. The importance of some molecular descriptors is demonstrated by comparing variations in the pEC_50_ or EC_50_ values with transformations in the values of molecular descriptors.

**Figure 4:**
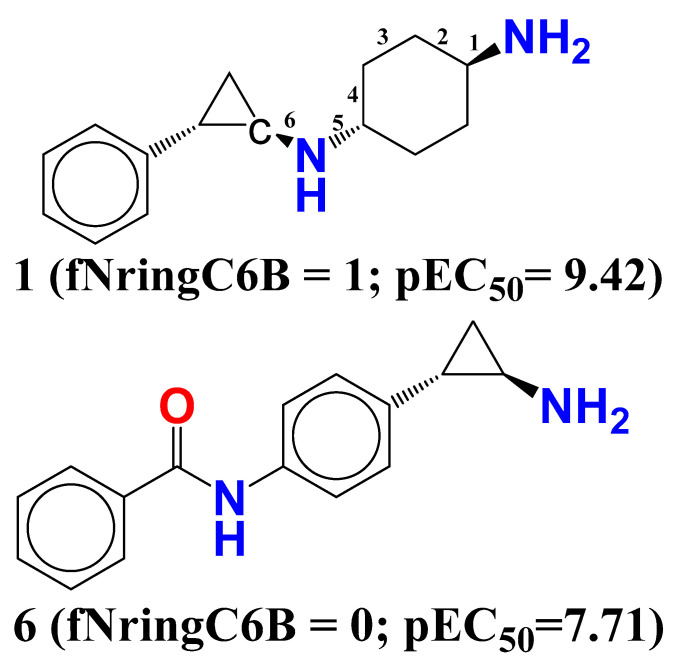
Illustration of molecular descriptor fNringC6B for the molecules 1 and 6 only.

**fNringC6B** (frequency of occurrence of ring carbon atom exactly at 6 bonds from nitrogen atom). This observation is supported by comparing compound **1** (fNringC6B = 1; pEC_50_= 9.42) with compound **6** (fNringC6B = 0; pEC_50_ = 7.71). Possibly, an increase in the value of fNringC6B to 1 for compound **6** enhanced its LSD1 inhibitory potency by about two hundred and twenty-two times (∆pEC_50_ = 2.22) (See Figure 4). 

This observation was also seen by comparing the subsequent pair of molecules: 17(_P_IC_50_ = 7.25, fNringC6B = 2) with 18(_P_IC_50_ = 7.21, fNringC6B = 0), 31(_P_IC_50_ = 7.04, fNringC6B = 4) with 32(_P_IC_50_ = 6.91, fNringC6B = 0), 52(_P_IC_50_ = 6.11, fNringC6B = 1) with 56(_P_IC_50_ = 5.88, fNringC6B = 0), etc. 

**Figure 5:**
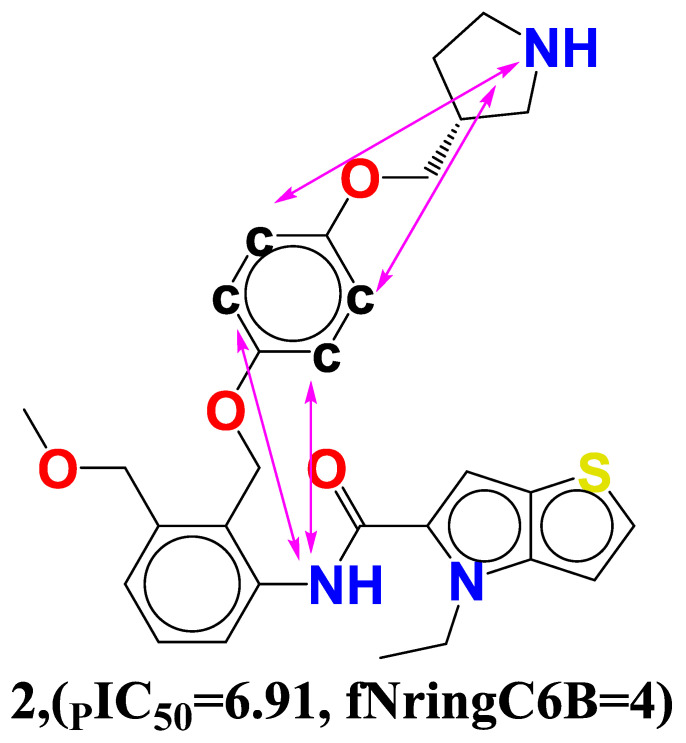
Depiction of the molecular descriptor **fNringC6B** in the compound **2**.

Vianello Paola et al. and colleagues also reported the synthesis of chemical 2 (4-ethyl-N-[3-(methoxymethyl)-2-[(4-[(3R)-pyrrolidin-3-yl] methoxyphenoxy) methyl] phenyl]- 4H- thieno [3,2-b] pyrrole-5-carboxamide) from the dataset (see Figure 5). The most efficient basic moiety was compound **2** with pyrrolidin-3-yl-methanol substituent, which had potent inhibitory activity against LSD1 (IC_50_ = 0.08570.02 M) according to structure–activity relationship studies. He went on to say that the polar interaction with two negatively charged regions of the LSD1 catalytic site is responsible for the compound **2**’s increased potency [10] (See Figure 5).

Thus, the present observation supports that the pyrrolidine ring enhances the polarity of the compound **2** that occurred exactly at 6 bonds. As a whole, the same feature has been captured in the QSAR model through the descriptor **fNringC6B**; therefore, QSAR results are complimentary with the reported findings. At the end, the QSAR model not only identified the polar nitrogen, but it also recognized the lipophilic carbon atom important for LSD1 inhibitory activity.

**lipo_ringS_8Bc** (Sum of partial charges of lipophilic atoms within 8 bonds from ring sulfur atom). The molecule with the better LSD1 inhibition might be obtained by enhancing the number of lipophilic atoms that accounted within 8 bonds from the sulfur atom. Just a four-fold amplification in the value of **lipo_ringS_8Bc** sufficed about $2\times 10^{3}$ fold more potent (∆pEC_50_ = 3.32) LSD1 inhibitor compound **4 (lipo_ringS_8Bc** = 0.19; pEC_50_ = 8.04) than compound **72** (**lipo_ringS_8Bc** = 0.05; pEC_50_ = 4.72). Several other pairs of compounds also support this observation: 31 (**lipo_ringS_8Bc** = −0.23; pEC_50_ = 7.046) with 32 (**lipo_ringS_8Bc** = 0; pEC_50_ = 6.917), 35 (**lipo_ringS_8Bc** = 0; pEC_50_ = 6.827) with 36 (**lipo_ringS_8Bc** = −0.28; pEC_50_ = 6.81), and 45 (**lipo_ringS_8Bc** = −0.207; pEC_50_ = 6.511) with 46 (**lipo_ringS_8Bc** = −0.231; pEC_50_ = 6.509).

**Figure 6:**
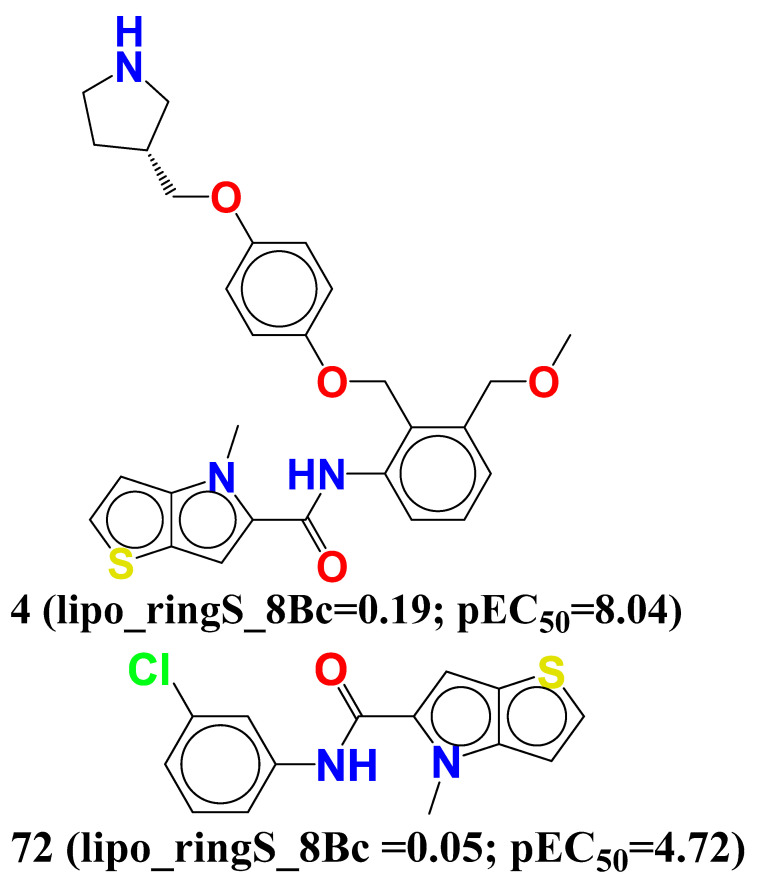
Depiction of molecular descriptor **lipo_ringS_8Bc** for the molecules 4, 72, and reported molecule 28186757 only.

Whence merely adding the number of carbon atoms is restricted (here average_molweight, i.e., molecular property average molecular weight, is with negative correlation) or inadequate, it is advisable to add electronegative atoms to the carbon atoms within 8 bonds from the ring sulfur to intensify the partial positive charge on the lipophilic atoms that boost up the LSD1 potency of the compound, respectively (see Figure 6).

Furthermore, a comparison of compound **4** to the previously reported molecule **28186757** suggests that increasing the amount of carbon atoms at the 8th position, specifically in the ether-containing carbon atom, will enhance the LSD1 inhibitory activity even more [10]. 

**Figure 7:**
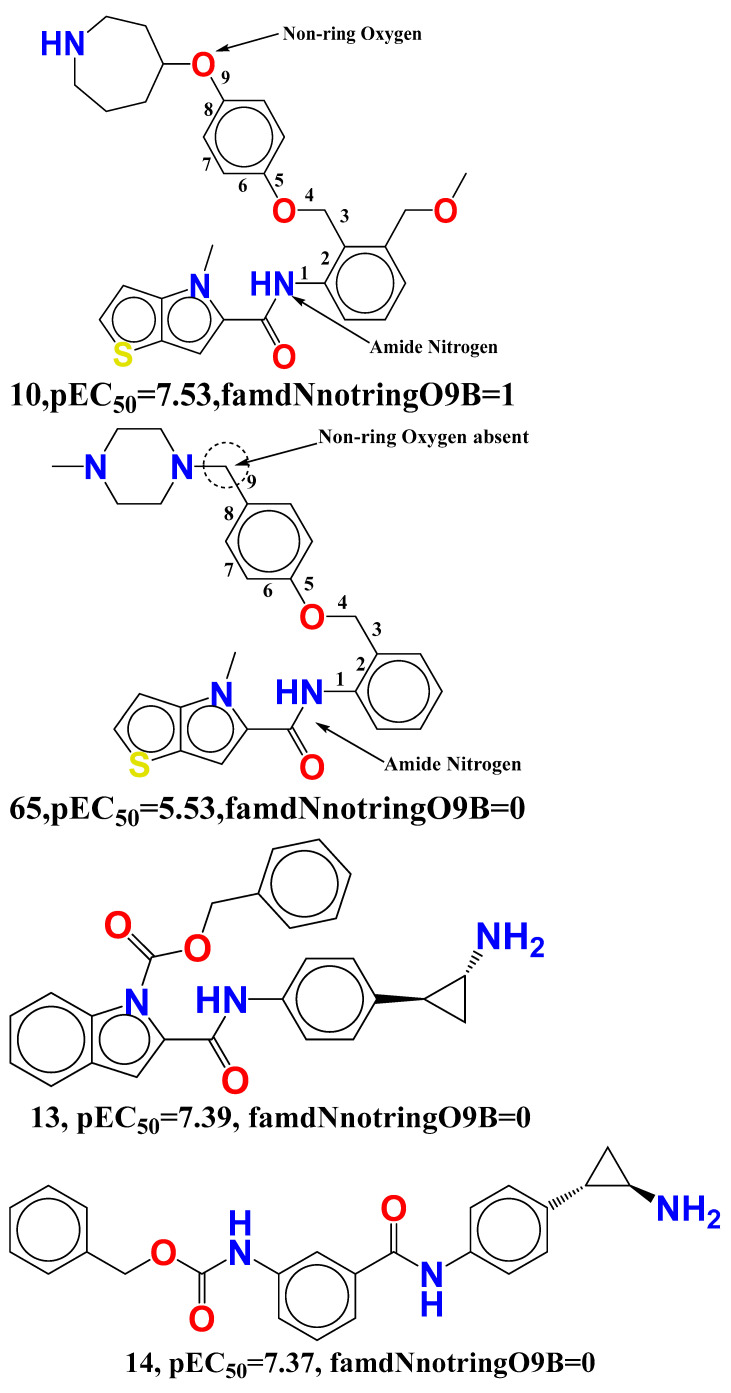
Pictorial depiction of molecular descriptor famdNnotringO9B for the molecules 10, 65, 13, and 14 only.

**famdNnotringO9B** (Frequency of occurrence of non-ring oxygen atom exactly at 9 bonds from the amide nitrogen) with a positive coefficient exhibit a direct correlation with LSD1 inhibitory potency. The four displayed compounds, **10**, **65**, **13,** and **14,** in Figure 7 illustrate the influence of the present molecular descriptor on the LSD1 inhibitory potency of the compound. It can be noted that, if the same non-ring carbon atom simultaneously occurred at one to eight bonds or more than 9 bonds from the amide nitrogen, then it is eluded during the calculation of famdNnotringO9B (see Figure 7).

Non-ring oxygen was detected exactly 9 links from the amide nitrogen in compound **10**, but the same oxygen was missing in compounds **65**, **13**, and **14**. This finding further supports the idea that the appropriate distance between the amide nitrogen and the non-ring oxygen is important for LSD1 inhibition. This helps to explain why molecules 10 and 65, 13, and 14 have different LSD1 inhibitory action. Instead, Vianello, Paola, and colleagues found that removing oxygen had only a little effect on the LSD 1 inhibitory function. This new discovery backs up the QSAR concept, emphasizing the significance of the oxygen atom in the 9th position from the amide nitrogen. In addition, Vianello Paola emphasized the importance of thieno [3,2-b]pyrrole-5-carboxamides as novel reversible inhibitors of the LSD1 receptor, noting that the same amide nitrogen was successfully detected as famdNnotringO9B in QSAR modelling. As a result, the QSAR results are consistent with the stated findings.

**Figure 8:**
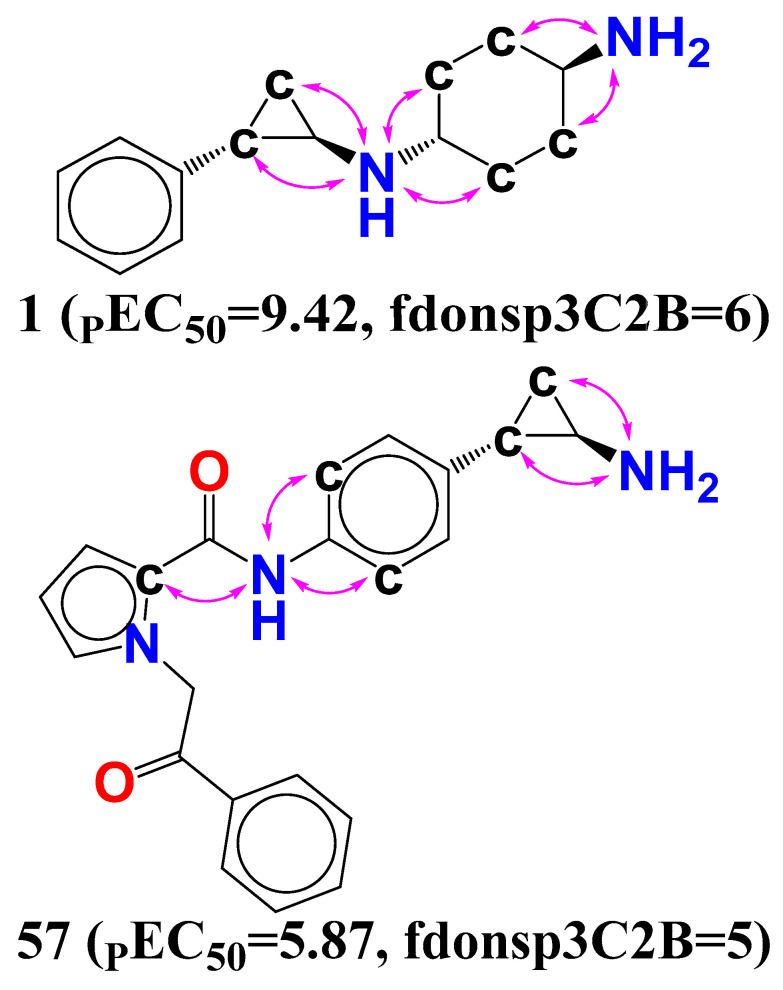
Pictorial display of molecular descriptor fdonsp3C2B for the molecules 1 and 57 only.

Another key chemical characteristic, **fdonsp3C2B** (frequency of occurrence of sp3 hybridised carbon atom exactly at 2 bonds from donor atom), is strongly linked with the reported bioactivity of LSD1 inhibitors. When comparing compound **1** to compound **57**, it can be shown that increasing the number of sp3 hybridised carbon atoms exactly at 2 bonds enhances the LSD1 inhibitory potency (see Figure 8). 

Furthermore, the same result holds true for a few other compounds: the most active compound **1** (_P_EC_50_ = 9.42, fdonsp3C2B = 6), as well as the compounds **2** (_P_EC_50_ = 8.17, fdonsp3C2B = 2), **3** (_P_EC_50_ = 8.10, fdonsp3C2B = 2), **4** (_P_EC_50_ = 8.07, fdonsp3C2B = 2), and **5** (_P_EC_50_ = 7.49, fdonsp3C2B = 2). The LSD1 inhibitory activity will be increased by 3.55 units if the value of the molecular descriptor fdonsp3C2B for the molecule 57 is increased from 2 to 6 (about a 35-fold increase in LSD1 inhibitory potency). Furthermore, sp3 hybridised carbon atoms should be added to boost LSD1 inhibitory activity, according to the current findings. Furthermore, increasing the amount of such sp3 hybridised carbons along the donor increases the electrical and hydrophobic interaction with the LSD1 receptor, showing lipophilicity.

**Figure 9:**
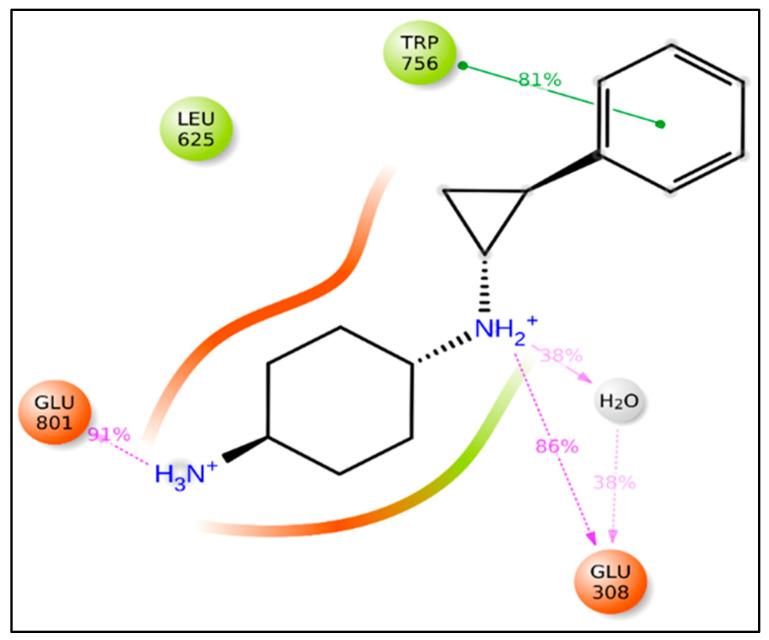
Depiction of the involvement of the molecular descriptor fdonsp3C2B in the LSD1-Compound 1 interactions during MD simulations.

Following that, it was discovered that during the MD modeling of compound **1** that the NH moiety, which acts as a donor with two bonds from the sp3 hybridized carbon atom (fdonsp3C2B), demonstrated significant hydrogen bonding with GLu308 (86 percent) and thus plays an important role in the stability of the LSD1–compound **1** complex. Furthermore, by including a water molecule, the same NH moiety created hydrogen bonds with a similar residue (GLu308), increasing the stability of the drug receptor complex. Furthermore, another NH2 substituent (91 percent) developed hydrogen bonding connections with the Glue801 residue, increasing the stability of the drug receptor complex (see Figure 9). This implies that the QSAR modelling has effectively identified certain important pharmacophores involved in the stability of the drug receptor complex, in addition to finding the many hidden structural elements crucial for LSD1 inhibition. As a consequence, the QSAR findings are entirely consistent with the molecular docking and MD simulation experiments.

No one chemical descriptor can explain the variation in inhibitory effectiveness of medicines in a dataset. The performance of the QSAR model is impacted by the synchronous effect of many molecular descriptors, some of which are not included in the QSAR models.

### 3.2. Molecular Docking

The molecular interaction of the five most active molecules with the LSD1 protein at the active site was investigated using molecular docking. The crystal structure of LSD1 was obtained using the RCSB protein data repository (https://www.rcsb.org/structure/2dw4, accessed on 24 May 2022) (PDB code: 2DW4). The full length of LSD1 comprises 852 amino acids with three key structure domains [11,12,13,14]: N-terminal Swi3-Rsc8-Moira domain (SWIRM domain, residues 172–270); C-terminal amine oxidase-like domain (AOL domain, residues 271–417 and 523–833); and central tower-like domain (Tower domain, residues 418–522). The SWIRM domain of LSD1 consists of six long α-helices (SWα1–6) and two stranded β-sheets (SWβ1–2), which regulates the chromatin remodeling and histone modification by taking part in protein–protein interactions.

**Figure 10:**
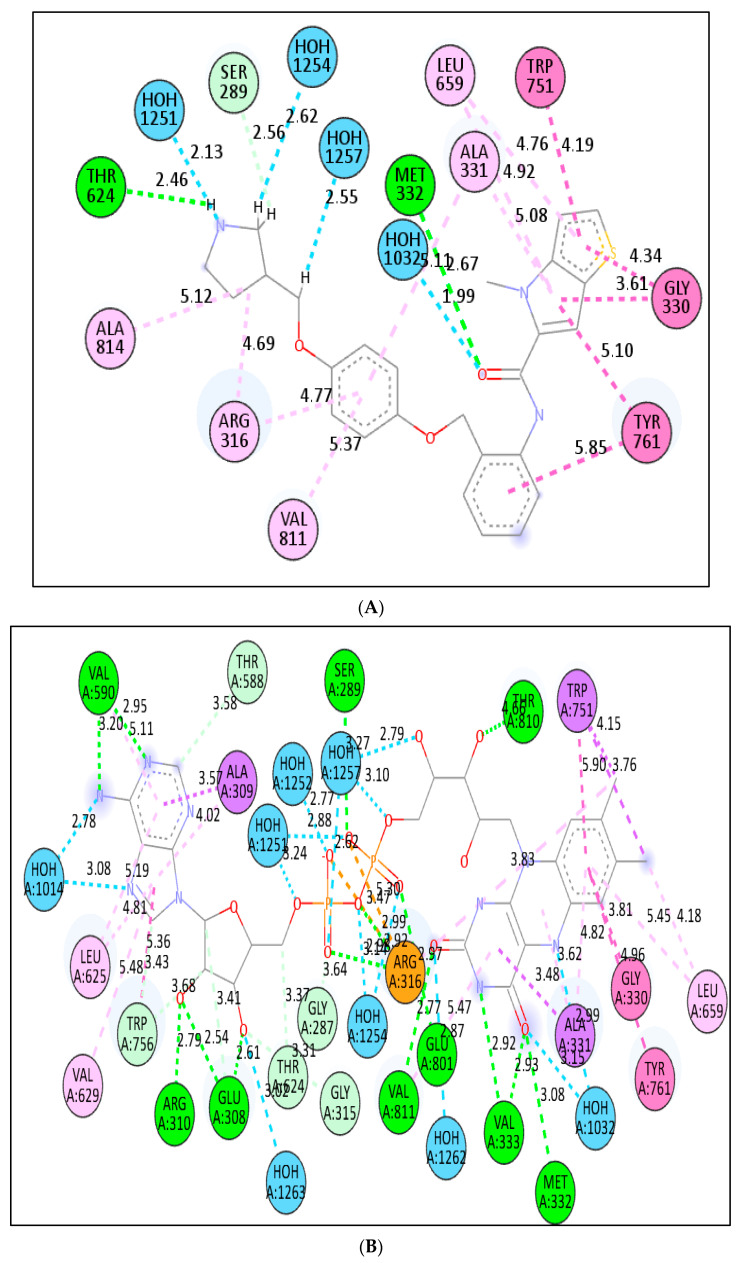
Presentation of the 2D interactions of compound **5** (**A**) and pdb-2dw4 ligand (**B**) with LSD1 receptor.

**Figure 11:**
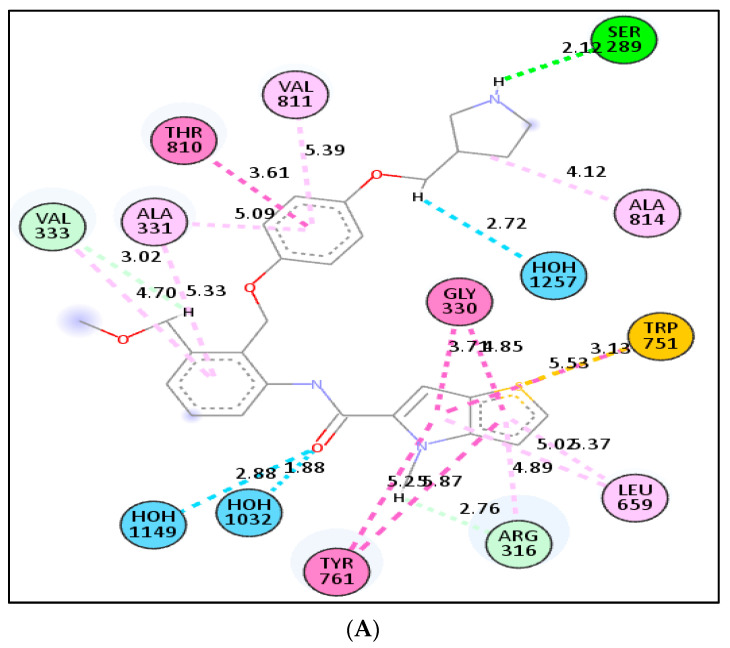

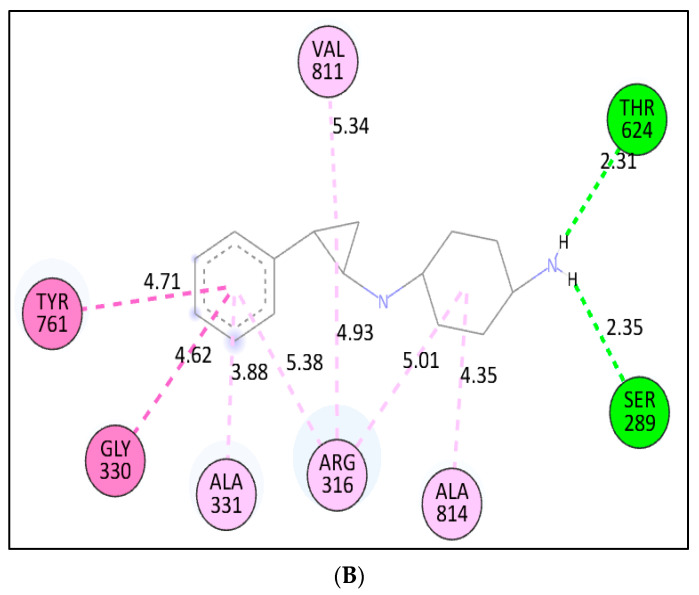
Presentation of the 2D and 3D interactions of compound **4** (**A**) and compound **1** (**B**) with LSD1 receptor.

**Figure 12:**
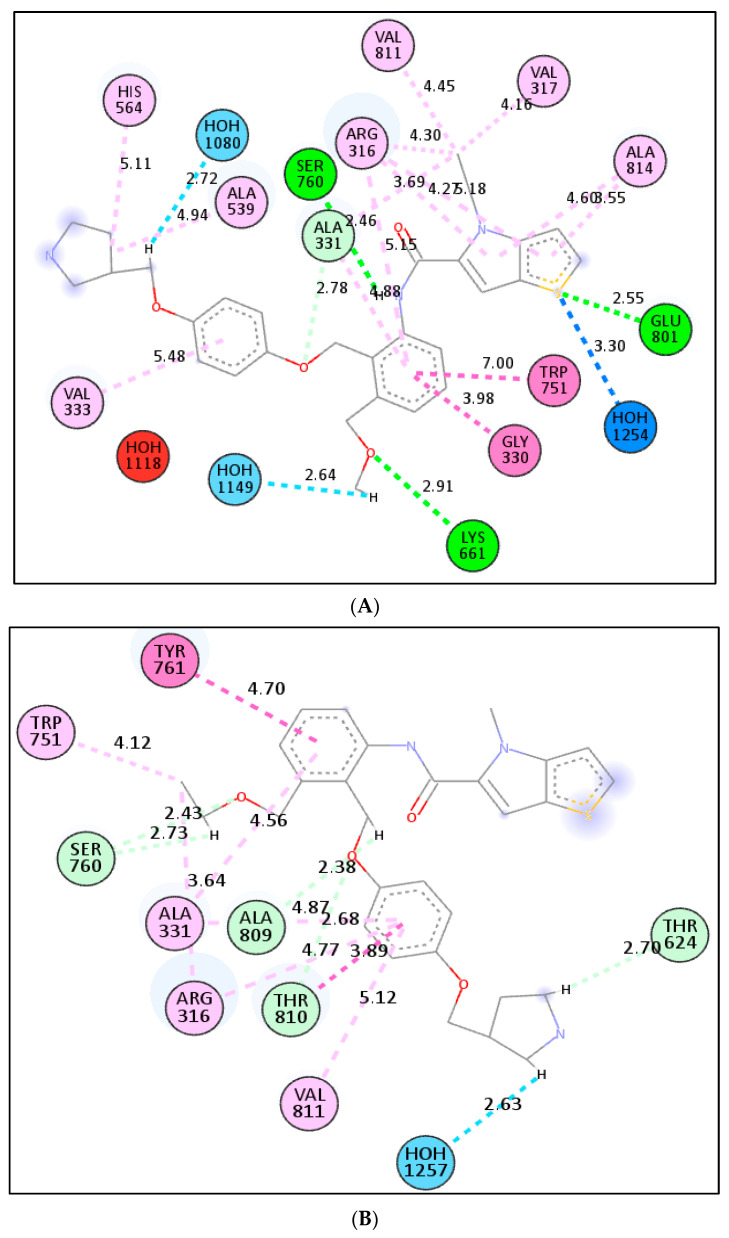
Presentation of the 2D and 3D interactions of compound **4** (**A**) and compound **1** (**B**) with LSD1 receptor.

**Figure 13:**
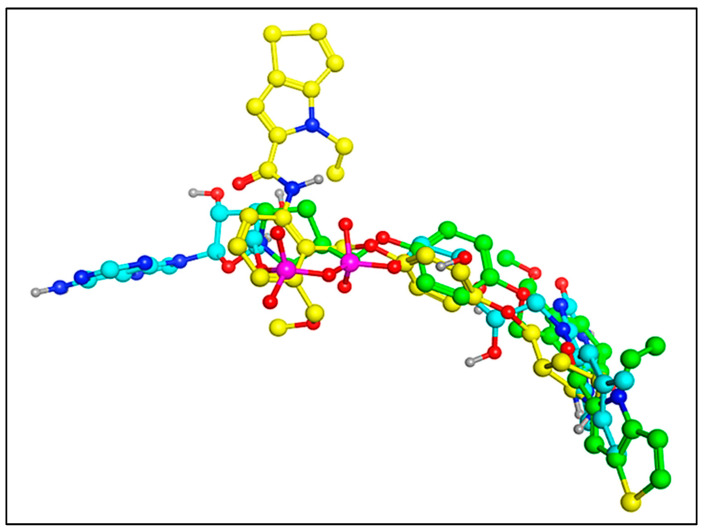
Comparison of the docked conformation of the compound **2** and **3** with the pdb-**2dw4** ligand. (**Green** colored—Comp **2**; **yellow** colored—Comp **3;s** and **cyan** colored—pdb-**2dw4** ligand).

**Figure 14:**
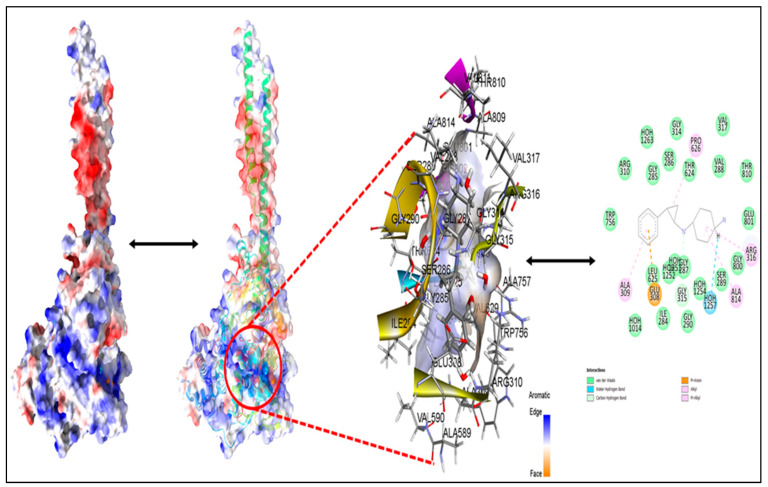
Best docked pose of compound **1** with LSD displaying 2D interaction plot on the left panel. Pink dashed lines indicate the Pi-Alkyl bond, and residues embedded in light green sphere indicate the involvement in Van der Waals interactions. On the canter panel, surface view of LSD displaying binding cavity of Compound **1** and right panel displaying the zoomed out binding pocket having amino acid residues surrounding the Compound **1**.

We investigated the probable interactions of inhibitors inside the active site of LSD1 to better understand the SAR and QSAR models of the five most active drugs. With an RMSD of 1.3618 A, the 2DW4 ligand was redocked into the LSD1 binding pocket. Because the accuracy of the docking results was determined by RMSD, this indicates that NRG Suite docking was able to effectively recognise the correct binding configuration (2.0). The docking scores for the five compounds, **1** (EC_50_ = 0.38 nm), **2** (EC_50_ = 6.7 nm), **3** (EC_50_ = 7.8 nm), **4** (EC_50_ = 8.4), **5** (EC_50_ = 18), and pdb-2dw4 ligand, were found to be −8.33(RMSD−1.38 A), −10.47(RMSD 1.82 A), −11.16(RMSD 1.32A), −11.10(RMSD 1.58A), −10.96(RMSD 1.13A), and −11.31(RMSD 1.36A) Kcal/mol, respectively, demonstrating that docking scores could predict ligand EC_50_ values. The 2D interactions for the five compounds were displayed in Figure 10, Figure 11, Figure 12, Figure 13 and Figure 14.

In terms of compound **5**’s low activity, the amide nitrogen forms a conventional hydrogen bond with the neutral non-polar amino acid residue MET332, a water–hydrogen bond with HOH1032, and a neutral polar amino acid residue with the pyrrolidine ring. THR624 forms a second hydrogen bond. With HOH1251, it creates a third water–hydrogen bond. TRP751, GLY330, LEU859, ALA331, TYR761, VAL811, ARG316, and ALA814, on the other hand, have been shown to form hydrophobic bonds with a thiene-pyrrole ring, a benzamide ring, a phenoxy ring, or a pyrrolidine ring (pi-pi T-shaped, amide-pi stacked, alky and pi-alkyl interactions). Despite the wide and flexible structure of compound five, the active conformation and compound-**5**–LSD1 complex were maintained via a variety of hydrophobic interactions and hydrogen bonding.

Compound **5** and compound **4** have similar interactions, although compound **4** is three times more powerful than compound **5**. In the structure, compound **5** has a folded shape, whereas compound **4** has an extended conformation akin to the pdb-2dw4 ligand. Within the active area of the LSD1 receptor, chemicals 5 and 4 are diametrically opposed. Except for one hydrophobic interaction with a TYR761 amino acid residue, the thien-pyrrole ring orientation was different. The QSAR models demonstrate the importance of the thiene-pyrrole ring for the reversible inhibition of the LSD1 receptor. The molecular descriptor **lipo_ringS_8Bc** indicates the importance of the Sum of partial charges of lipophilic atoms within 8 bonds from ring sulfur atoms. With TRP751, compound **4** (lipo ringS 8Bc= −0.1869) made more than eight types of hydrophobic connections and one pi-sulphure interaction, whereas compound **5** (lipo ringS 8Bc = −0.2319) made seven hydrophobic contacts (See Figure 11A,B). The difference in the reactivity of these compounds was linked with the occurrence of positively charged lipophilic atoms. The present observation indicates that the decrease in the negative charge promotes more hydrophobic contacts in the compound **4**. Furthermore, in compound **1** (lipo ringS 8Bc = 0), partial positive charges are zero, underlining the observation of declining negative charges and intensifying positive charges within the thiene-pyrrole ring, which promotes better hydrophobic contact with the LSD1 receptor. The compounds **3** (lipo_ringS 8Bc = −0.1869) and 2 (lipo_ringS 8Bc = −0.2339) showed the same behavior. Finally, QSAR analysis was successful in uncovering latent pharmacophoric characteristics that determine not only the LSD1 inhibitory action of these compounds, but also their binding pattern. As a result, the molecular docking analysis results are entirely congruent with the QSAR findings.

Moreover, compound **2** (EC_50_ = 6.7 nm), was marginally more potent than compound **3** (EC_50_ = 7.8 nm). The 2D interactions for compounds **2** and **3** show that compound **2** produced three standard hydrogen bonding contacts with SER760, LYS661, ARG316, and GLU801, but compound **3** did not form any conventional hydrogen bonding interactions with SER760, ALA809, THR810, or HOH1257. Moreover, compound **2** executed more than 11 different hydrophobic contacts with the HIS564, ALA539, VAL333, GLY330, TRP751, VAL811, VAL317, ALA814, etc. Moreover, the thiene-pyrrole ring in the compound didn’t contribute in any of the hydrophobic contact, but it aligned over the solvent accessible surface area of the LSD1 receptor (See Figure 12A,B). Furthermore, when the conformations of compounds **2** and **3** are compared to the pdb-2dw4 ligand, it is clear that compound **2** aligns and superimposes entirely along the docked conformation of the pdb ligand. Following that, in compound **3**, the thiene-pyrrole ring aligns vertically in the receptor (LSD1) binding pocket, which is completely different from the bioactive conformation of the pdb ligand and could explain the difference in potency between these compounds (see Figure 13, green-comp-2, yellow-comp-3, and cyan-pdb-2dw4 ligand). The benzene ring connected to the thiene-pyrrole ring by amide linkage in compound **3** contains a bulky substituent (methoxy ethyl) compared to compound **2** (methoxy methyl), which may have hampered compound **3**’s ability to achieve the same bioactive conformation as the pdb ligand. This helps to explain the differences in potency and binding affinity for the LSD1 receptor.

The compound **2** (_P_IC_50_ = 8.174, **fdonsp3C2B** = 2, lipo_ringS 8Bc = −0.2339) and 3 (_P_IC_50_ = 8.174, **fdonsp3C2B** = 2, lipo_ringS 8Bc= (_P_IC_50_ = 8.174, **fdonsp3C2B** = 2, lipo_ringS 8Bc = −0.2339) differed from the compound **1** (_P_IC_50_ = 9.42, **fdonsp3C2B** = 6, lipo_ringS 8Bc = 0) in terms of two descriptors: **fdonsp3C2B and** lipo_ringS 8Bc. Compound **3** can’t form hydrogen bonds or hydrophobic interactions with the receptor because of its altered orientation. The amide donor produced hydrogen bonds with the SER760 residue in compound **2**, whereas another donor nitrogen of the terminal pyrrolidine ring aligned over the solvent accessible surface region, and the sp3 hybridised carbon atom made hydrophobic interactions with the receptor. In the QSAR model, the same feature was captured. In addition, the thiene-pyrrole ring sulphure atom formed conventional hydrogen bonds with the ARG316 and GLU 801 residues, as well as a water–hydrogen link with the HOH1254 residue. The relevance of the thiene-pyrrole sulphure atom, which was captured in the QSAR model as lipo ringS 8Bc descriptors, is highlighted by this observation. Furthermore, the lipophilic carbon atoms in the benzene ring of compound **2** connected to the thiene-pyrrole ring via amide linkage generate distinct hydrophobic interactions with the receptor. This finding emphasises the significance of positively charged lipophilic carbon atoms in drug receptor interactions. Thus, QSAR modelling was successful in identifying the features required to improve binding affinity, and the results were in perfect agreement with the molecular docking data. In addition, comparison with the most active compound **1**(PIC50 = 9.42, fdonsp3C2B = 6, lipo ringS 8Bc = 0) indicated the importance of the lipophilic, as well as the electronic properties required for binding affinity and, ultimately, LSD1 receptor inhibition.

The docking results revealed that the descriptors, **fdonsp3C2B** and **lipo_ringS_8Bc**, played important roles in the inhibition of the LSD1 receptor, which was consistent with the QSAR findings.

### 3.3. Molecular Dynamic Simulations

During the simulation, monitoring the protein’s RMSD can provide insight into its structural conformation. The RMSD analysis can identify if the fluctuations at the end of the simulation are centred on some thermal average structure if the simulation has equilibrated. For tiny, spherical proteins, changes on the order of 1–3 are perfectly acceptable. Larger changes, on the other hand, imply that the protein is significantly changing form during simulation. It’s also crucial that your simulation converges, which means the RMSD values settle around a fixed number. If the average RMSD of the protein is still increasing or dropping at the end of the simulation, your system has not equilibrated, and your simulation may not be lengthy enough to do a thorough analysis. Ligand RMSD (right *Y*-axis): the ligand RMSD (right *Y*-axis) shows how stable the ligand is in relation to the protein and its binding pocket.

When the protein–ligand complex is aligned on the reference protein backbone first, and then the RMSD of the ligand-heavy atoms is measured, the RMSD of the ligand is plotted. If the observed values are significantly greater than the RMSD of the protein, the ligand has most likely diffused away from its initial binding site. 

**Figure 15:**
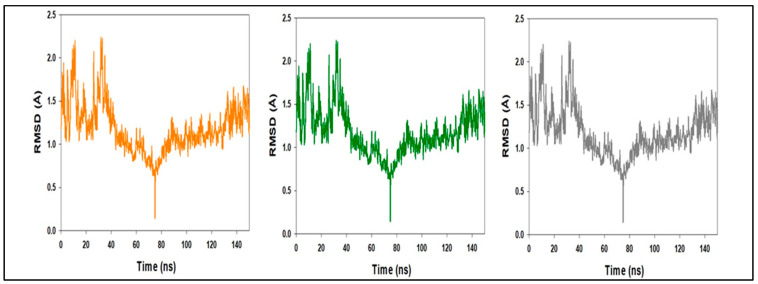
MD simulation trajectory analysis of Root Mean Square Divisions (RMSD) of compound **1** bound with LSD; 150 ns time frame in triplicate displayed.

The above-mentioned diagram is the triple run result of Root Mean Square Divisions (RMSD) MD simulation trajectory analysis. The RMSD plot of the LSD–compound **1** complex (Figure 3) indicates that the complex stabilizes at about 20 ns. After that, for the length of the simulation, swings in RMSD values for target remain within 0.5, which is absolutely acceptable. The ligand fit-to-protein RMSD values fluctuate within 0.7 Angstrom after they have been equilibrated. These findings indicate that the ligands stayed firmly connected to the receptor’s binding site throughout the simulation period. The RMSD values for ligand fit to protein do not change much during the simulation duration, showing that the ligands remain securely attached to the receptor’s binding site, as shown in Figure 15. 

**Figure 16:**
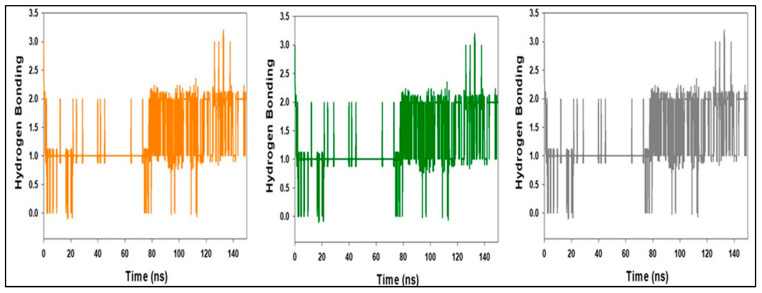
MD simulation trajectory analysis of Hydrogen-bonding (H-bonding) of compound **1** bound with LSD 150 ns time frame in triplicate displayed.

Figure 16 shows the average hydrogen bonds established throughout the 150 ns triple simulation between compound **1** and the various proteins. From 0 to 150 ns, an average of four hydrogen bonds are observed for LSD, and the same is true for triple MD simulations of compound **1** and LSD (Figure 16). Throughout the simulation, two hydrogen bonds were established, as shown by the 2D ligand binding figure. The number of hydrogen bonds between LSD and compound **1** has increased, making the binding stronger and more robust over simulation. 

**Figure 17:**
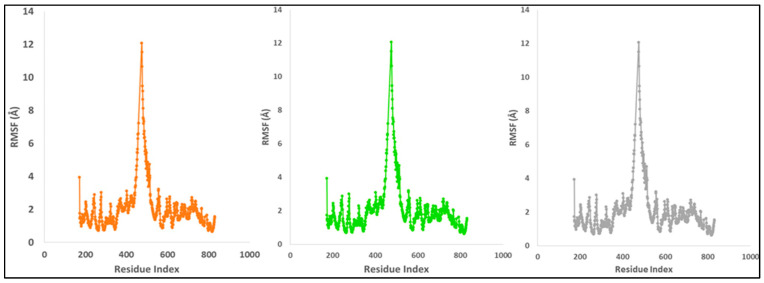
MD simulation trajectory analysis of Root Mean Square Fluctuations (RMSF) of compound **1** bound with LSD with their triplicate runs.

On the RMSF plot, peaks represent portions of the protein that fluctuate the most during the simulation. Protein tails (both N- and C-terminal) typically change more than any other part of the protein. Alpha helices and beta strands, for example, are usually stiffer than the unstructured component of the protein and fluctuate less than loop sections. According to MD trajectories, the residues with greater peaks belong to loop areas or N- and C-terminal zones (Figure 17). Although there is some instability between 400 and 600 residues, the stability of ligand binding to the protein is demonstrated by stable RMSF values of binding site residues.

**Figure 18:**
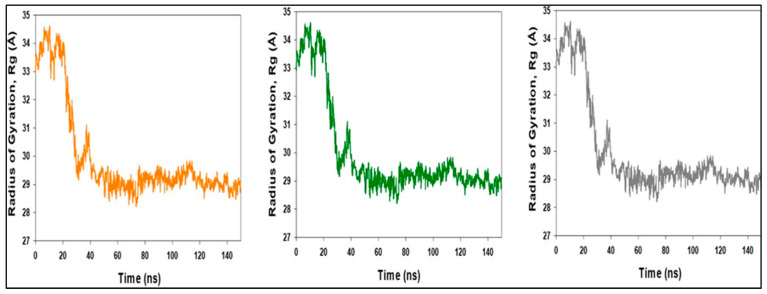
MD simulation trajectory analysis of Radius of Gyration (RoG) of compound **1** bound with LSD with their triplicate runs.

The compactness of proteins is measured by the radius of gyration. The Radius of Gyration of LSD proteins bound to compound **1** was reduced (Figure 18). Compound **1** bonded to the protein targets posthumously in the binding cavities and plays a substantial role in the stability of the proteins, according to the overall quality analysis using RMSD and Rg.

**Figure 19:**
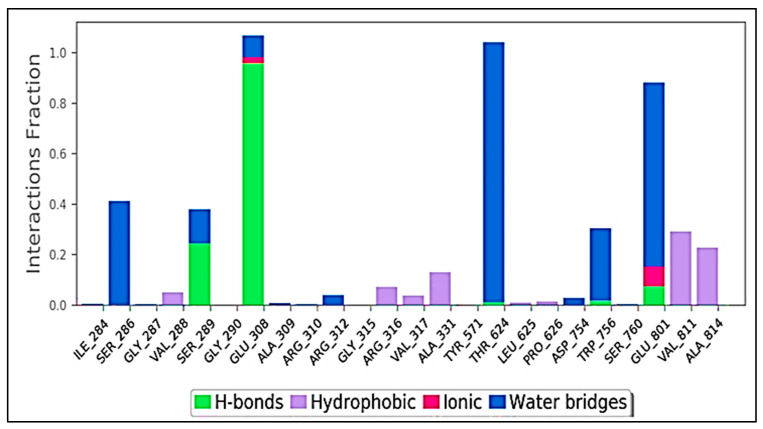
Protein-ligand contact histogram (H-bonds, Hydrophobic, Ionic, Water bridges) of LSD and compound **1**.

Protein interactions with the ligand can be detected throughout the simulation. These interactions can be categorized and summarized by type, as shown in the graphs below. The four types of protein–ligand interactions (or ‘contacts’) are hydrogen bonds, hydrophobic, ionic, and water bridges. The ‘Simulation Interactions Diagram’ panel in Maestro can be used to analyse the subtypes of each interaction type. Over the course of the trajectory, the stacked bar charts are standardised; for example, a value of 0.7 indicates that the specific interaction is maintained for 70% of the simulation duration. Values exceeding 1.0 are possible because some protein residues may have several interactions with the same subtype of ligand. The majority of the important ligand–protein interactions found by MD are hydrogen bonds and hydrophobic interactions, as shown in Figure 19. In terms of H-bonds, the LSD–compound **14** complex residues VAL 4288, GLY 290, TYR 571, ASP 754, and SER 760 are the most essential. Over the course of the trajectory, the stacked bar charts were standardised; for example, a value of 1.0 signifies that the specific interaction was maintained for 100% of the simulation duration. Values exceeding 1.0 are possible because some protein residues may have several interactions with the same subtype of ligand.

**Figure 20:**
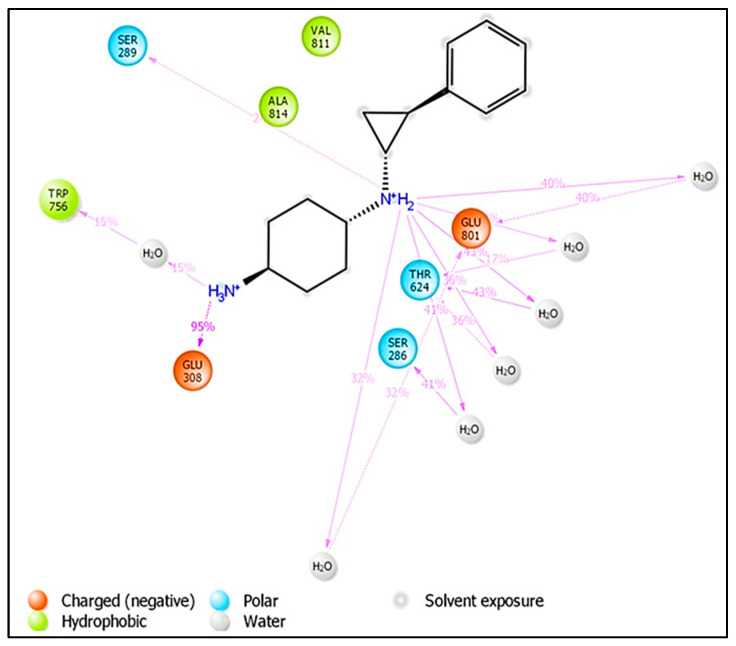
Ligand atom interactions with the protein residues LSD-compound **1**.

Figure 20 depicts individual ligand atom interactions with protein residues. Interactions that last more than 30.0 percent of the simulation time (0.00 through 150.0 ns) in the chosen trajectory are shown.

**Figure 21:**
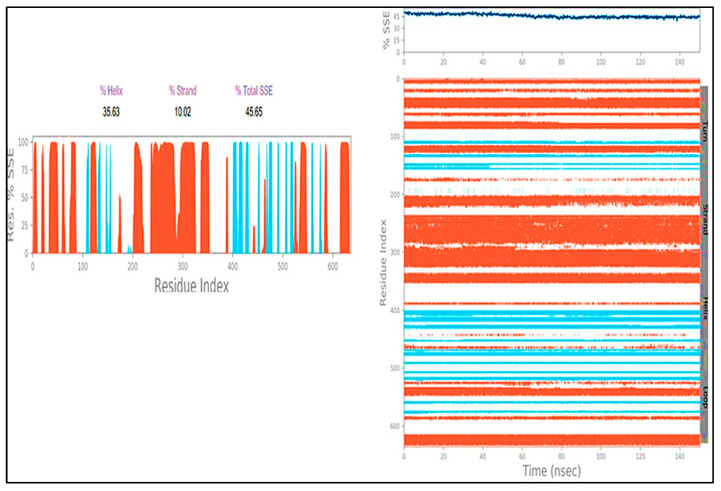
Secondary Structure element distribution by residue index throughout the protein structure. Red indicates alpha helices, and blue indicate beta-strands of; LSD-compound **1**.

The presence of protein secondary structural elements (SSE) such as alpha helices and beta strands is checked throughout the simulation to guarantee that they are not present. The plot above shows the distribution of SSE by residue index over the entire protein structure, and it includes all residues. The graphs at the bottom illustrate the evolution of each residue and its SSE assignment throughout the experiment, in contrast to the charts below, which show a summary of the SSE composition for each trajectory frame during the simulation (as shown in Figure 21). 

**Figure 22:**
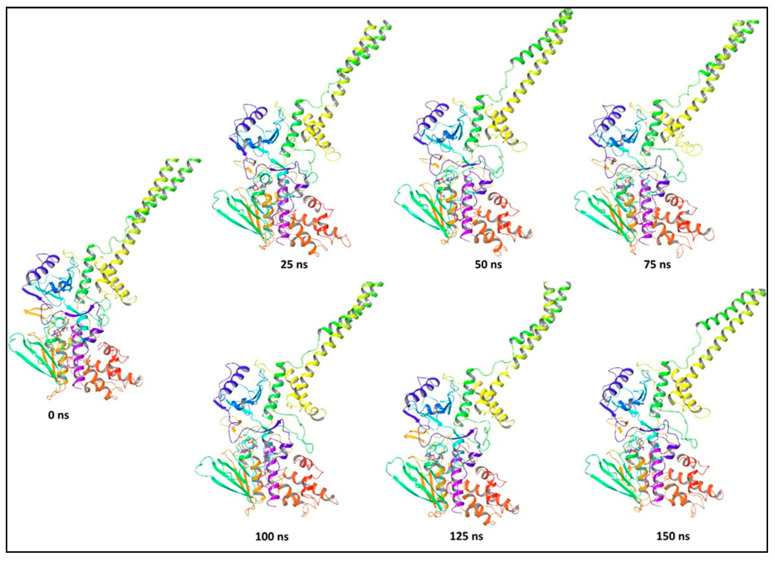
Stepwise trajectory analysis for every 25 ns displaying the protein and ligand conformation during 150 ns of simulation of LSD-compound **1**.

In comparison to the 0 ns structure, the positional change was obvious in the stepwise trajectory analysis of every 25 ns of compound **1** simulation with LSD (Figure 22). In order to achieve conformational stability and convergence, the ligand, compound **1**, was discovered to exhibit structural angular mobility at the end frame. 

The ligand torsions graphic depicts the conformational evolution of each rotatable bond (RB) in the ligand throughout the simulation trajectory (0.00 through 150.00 ns). The top panel shows a two-dimensional schematic of a ligand with color-coded rotatable bonds. Each rotatable bond torsion is accompanied with a dial plot and a bar plot of the same colour. The structure of the torsion during the simulation is depicted by dial (or radial) charts. The simulation begins in the radial display’s centre, and the time evolution is plotted radially outwards.

**Figure 23:**
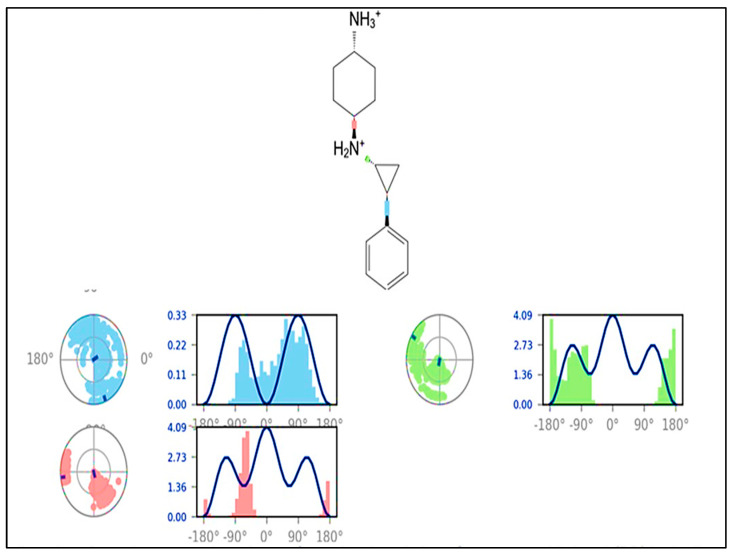
Ligand torsion profile of LSD-compound **1** displayed during 150 ns of simulation 1.

In the bar charts, which summarize the data from the dial plots, the probability density of the torsion is shown. If torsional potential data is provided, the graphic also displays the potential of the rotatable bond (by summing the potential of the related torsions). The potential values are given in kcal/mol and are displayed on the chart’s left *Y*-axis. The correlations between the histogram and torsion potential can reflect the conformational strain that the ligand undergoes in order to maintain a protein-bound shape (See Figure 23).

**Figure 24:**
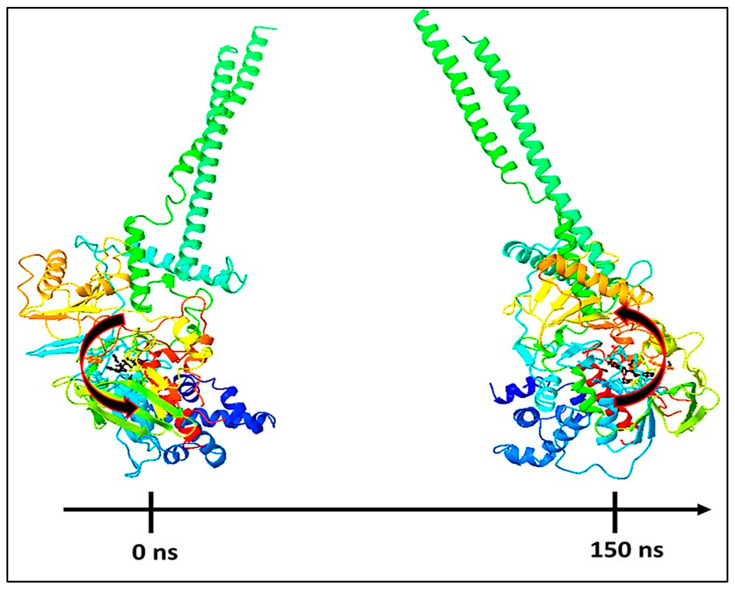
MMGBSA trajectory (0 ns, before simulation and 150 ns, after simulation) exhibited conformational changes of compound **1** upon binding with the proteins, LSD, the arrows indicating the overall positional variation (movement and pose) of compound **1** at the binding site cavity.

Molecular Mechanics Generalized Born and Surface Area (MMGBSA) calculations. The MMGBSA method is often used to determine the binding energy of ligands to protein molecules. The binding free energy of each protein–compound **1** complex was calculated, as well as the influence of the other non-bonded interactions energies (Table 1). The binding energy of ligand compound **1** with LSD is −59.78 kcal/mol. Gbind is governed by non-bonded interactions such as GbindCoulomb, GbindCovalent, GbindHbond, GbindLipo, GbindSolvGB, and GbindvdW. The GbindvdW, GbindLipo, and GbindCoulomb energies contributed the most to the average binding energy across all types of interactions. These conformational alterations result in improved binding pocket acquisition and engagement with residues, resulting in increased binding energy and stability. Thus, the binding energy obtained from docking results was well justified by MM-GBSA calculations. Furthermore, the last frame (150 ns) of MMGBSA displayed the positional change of compound **1** as compared to the 0 ns trajectory, indicating a better binding pose for best fitting in the protein’s binding cavity (see Figure 24).

## 4. Materials and Methods

### 4.1. Preparation of Data Sets/Modeling Set Preparation from ChEMBL Data

**Figure 25:**
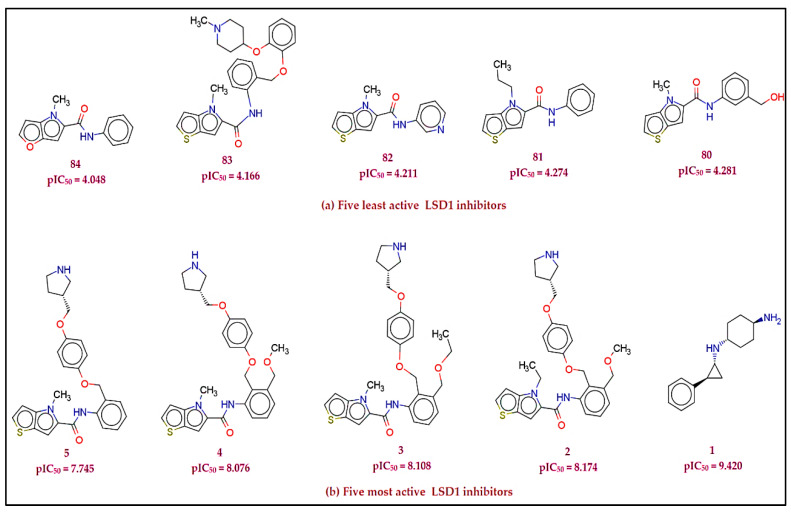
Representative (**a**) five least active and (**b**) five most active LSD1 inhibitors from the selected data set.

Only compounds having experimental LSD1 inhibitory potency tested against a range of LSD1 inhibition assays were used in the ChEMBL [9] database. A limited data set of 84 LSD1 inhibitors with accurate EC_50_ values (0.38–89500 nM) was created from a crude dataset of 191 compounds with experimental EC_50_ values after removing structural duplicates, multi-component compounds or salts, and compounds with imprecise EC_50_ values. The EC_50_ values in nanomolar (nM) units were converted to molar units first (M). For the sake of data set handling, EC_50_ (M) values for each molecule were transformed to pEC_50_ (pEC_50_ = −logEC_50_). SMILES notations for all 84 substances with experimental EC_50_ and pEC_50_ values are listed in Table S1 in Supplementary. Figure 25 shows a representative example of the five least active and five most active LSD1 inhibitors.

### 4.2. Calculation of Molecular Descriptors and Objective Feature Selection (OFS)

Using Open Babel 3.1, the SMILES notations were translated to 3D structures [15]. The most stable conformation is found in the geometry optimized molecule. As a result, calculating molecular descriptors on a dataset of optimized molecules assures that all physico-chemical attributes for all molecules in the dataset are uniform. Prior to calculating molecular descriptors, all of the compounds in the current dataset were optimized using TINKER (force field MMFF94). An appropriate calculation of many molecular descriptors is required in QSAR analysis to improve mechanistic understanding. A huge collection of more than 30,000 unique 1D- to 3D-molecular descriptors may be found in PyDescriptor, a PyMOL plugin [16]. Data trimming was performed to prevent the risk of overfitting due to noisy duplicated descriptors. Then, using QSARINS-2.2.4 [17], objective feature selection (OFS) was used to exclude near-constant, constant, and significantly inter-correlated (|R| > 0.90) molecular descriptors. Despite the fact that only 1733 molecular descriptors were accepted into the contracted molecular descriptor pool, it nevertheless has a wide range of descriptors that cover a wide chemical spectrum.

### 4.3. Splitting of the Data Set Molecules into Training and External Sets and Subjective Feature Selection

To avoid information leaking, it is critical to divide the entire data set into training and prediction sets with correct configuration and sizes prior to rigorous subjective feature selection [18]. To avoid bias, the entire data set was arbitrarily divided into two sets: training (an 80%, or 67 molecules) and prediction (20%, or 17 molecules). The sole objective of a training set is to select an acceptable number of molecular descriptors for developing QSAR models, whereas the prediction set is used to validate these models externally (Predictive QSAR). The genetic algorithm-reinforced multilinear regression (GA-MLR) method, as implemented in QSARINS-2.2.4, was used to pick acceptable descriptors using Q^2^_LOO_ as a fitness parameter for subjective feature selection.

**Figure 26:**
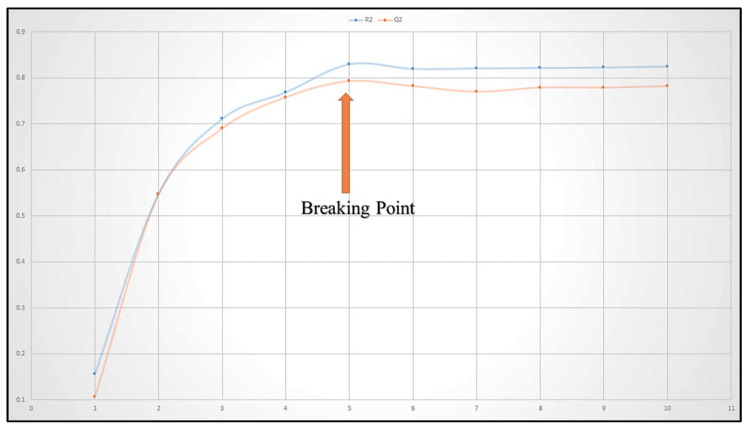
Depiction of Plot for the number of descriptors against the Coefficient of Determination R^2^ and Leave-One-Out Coefficient of Determination Q^2^ to identify the optimum number of descriptors.

To construct a good QSAR model, it is critical to avoid overfitting and to choose an appropriate number of molecular descriptors in order to provide satisfactory interpretability. As a result, a graph of the number of molecular descriptors (*X*-axis) involved in the models against R^2^tr and Q^2^_LOO_ values (*Y*-axis) has been plotted in the current communication to achieve breaking point, with the number of molecular descriptors corresponding to the breaking point being an optimum number of descriptors in QSAR model building. Because the graph in Figure 3 shows a breaking point at five variables, QSAR models with more than five descriptors were eliminated (See Figure 26).

### 4.4. Model Development and Validation

The robustness of the created models was determined using a variety of validation criteria reported in the literature. Internal predictability and statistical quality of the developed model were tested using parameters such as the coefficient of determination (r^2^), leave-one-out cross-validation (Q^2^_LOO_), and leave-many-out cross-validation to achieve this (Q^2^ _LMO_). In addition, for each developed model, the standard error of estimate(s) was defined. For the given QSAR models for the stated dataset, RMSE (Root Mean Squared of Errors) for the training (RMSE_TR_) and external prediction sets (RMSE_ext_) that denote the complete error of the model that was predicted as an extra portion of the accuracy [5,18] were used.

The QUIK rule (Q Under the Influence of K) was used to examine the inter-correlation between descriptors. To reduce inter-correlation among descriptors, the QUICK rule was set to 0.05. The fit of the randomly reordered Y-data was checked using Y-randomization with 2000 iterations to ensure the trustworthiness of the created QSAR model. The dependent variables (pEC50 value) of the training set were shuffled, and new coefficients of determination were produced for the randomization of the constructed QSAR model. The new models’ coefficients of determination are significantly low, indicating that the reported model in this QSAR research was not acquired by chance correlation [19].

All models were externally validated using the following validation criteria: r^2^ext (external determination coefficient), Q^2^_F1_, Q^2^_F2_, Q^2^_F3_, Concordance Correlation Coefficient (CCC), CCC_ex_, r^2^m, and r^2^m. The R^2^m (overall) parameter penalizes a model when there are big disparities between observed and predicted values of all the compounds in the collection (considering both training and test sets). The difference between the values of the expected and the resultant experimental activity data was assessed using the r^2^m (pEC_50_ value). It has been suggested that the observed value for the r^2^m should be lower than 0.2 if the r^2^m value is more than 0.5. To validate model reliability and robustness, all QSAR models were examined for validation parameters such as Golbraikh and Tropsha’s criterion.

In general, the created QSAR model’s predictive ability is determined by how well the anticipated value matches the observed (experimental biological activity) value. Even the presence of a single outlier reduces the generated QSAR model’s prediction ability. Following that, we attempted to identify the outliers based on compound with a considerably high residual value in GA-MLR QSAR models. Furthermore, by comparing the predicted value to the standardized residual values, we were able to identify the outlier compounds. Similarly, the leverage effect in Williams’ plot revealed structural variation in database compounds. The created QSAR model’s applicability domain is determined by combining the leverage and standard residuals [20,21,22,23].

### 4.5. Molecular Docking Analysis

**Figure 27:**
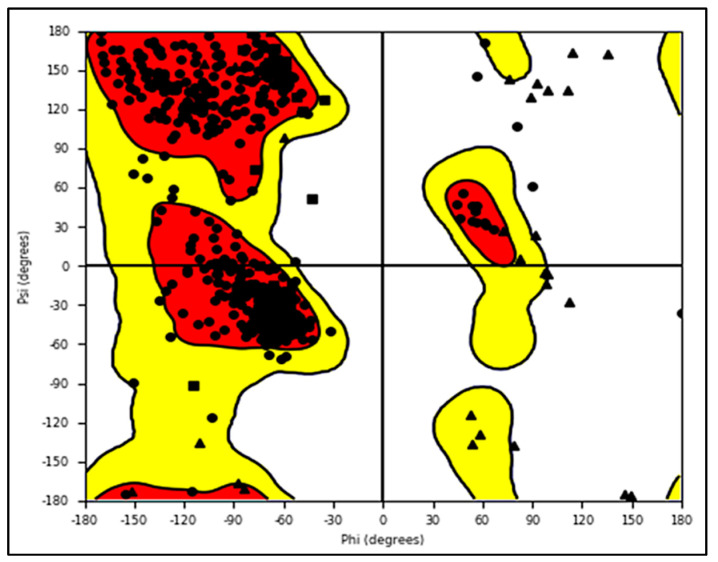
Ramchandran plot for LSD1 receptor.

The protein data bank (https://www.rcsb.org/structure/2DW4, accessed on 24 May 2022) was used to obtain the pdb file for the LSD1 receptor. The pdb 2dw4 [24] was chosen because of its X-ray resolution and sequence completion. The health of the protein was evaluated before actual docking simulations by plotting Ramchandran’s plot [25] (See Figure 27). For docking analysis, the optimized protein is acceptable. Although all of the compounds were docked into the active site, the docking pose for the most active compound 1 as a representative has been shown below for convenience.

The software NRGSuite [26] was utilized for molecular docking analysis. This open-source software is accessible as a PyMOL plugin (www.pymol.org, accessed on 7 July 2022). With the help of FlexAID [27], it can detect the surface cavities in a protein and use them as target-binding sites for docking simulations. It models ligand and side-chain flexibility, as well as covalent docking, and employs a genetic algorithm for conformational search. To gain the best performance using NRGsuite, we used a flexible-rigid docking technique with the following default settings: Side chain flexibility—no; ligand flexibility—yes; ligand pose as reference—no; constraints- no; HET groups- included water molecules; van der Walls permeability—0.1; solvent types—no type; number of chromosomes—1000; number of generations—1000; fitness model—share; reproduction model—population boom; number of TOP complexes—5. The native ligand, a known tranylcypromine inhibitor of LSD1 [24], was used to validate the docking technique for molecular docking.

### 4.6. Molecular Dynamic Simulation 

Desmond, a package from Schrödinger LLC [28], was used to simulate molecular dynamics for 150 nanoseconds. Docking experiments provided the earliest step of protein and ligand complexes for molecular dynamics simulation. In static settings, Molecular Docking Studies can predict the ligand binding state. Because docking provides a static view of a molecule’s binding pose in a protein’s active site [29], MD simulations tend to compute atom movements over time by integrating Newton’s classical equation of motion. The ligand binding status in the physiological milieu was predicted using simulations [30,31]. 

Protein Preparation Wizard or Maestro was used to preprocess the protein–ligand complex, which included complex optimization and minimization. The System Builder tool was used to prepare all of the systems. TIP3P was chosen as a solvent model with an orthorhombic box (Transferable Intermolecular Interaction Potential 3 Points). In the simulation, the OPLS 2005 force field was used [32]. Counter ions were added to the models to make them neutral. A total of 0.15 M salt (NaCl) was added to replicate physiological circumstances. For the entire simulation, the NPT ensemble with 300 K temperature and 1 atm pressure was chosen. Before the simulation, the models were loosened. After every 100 ps, the trajectories were saved for analysis, and the simulation’s stability was determined by measuring the root mean square deviation (RMSD) of the protein and ligand over time.

### 4.7. Molecular Mechanics Generalized Born and Surface Area (MMGBSA) Calculations

During MD simulations of LSD complexed with complex 1, the binding free energy (Gbind) of docked complexes was calculated using the premier molecular mechanics generalized born surface area (MM-GBSA) module (Schrodinger suite, LLC, New York, NY, USA, 2017-4). The binding free energy was calculated using the OPLS 2005 force field, VSGB solvent model, and rotamer search methods [16,17,18]. After the MD run, 10 ns intervals were used to choose the MD trajectories frames. The total free energy binding was calculated using Equation (1):

∆Gbind = Gcomplex − (Gprotein + Gligand) (1)

where, ∆Gbind = binding free energy, Gcomplex = free energy of the complex, Gprotein = free energy of the target protein, and Gligand = free energy of the ligand. The MMGBSA outcome trajectories were analyzed further for post-dynamics structural modifications. 

## 5. Conclusions

Pharmacophoric traits responsible for improved LSD1 inhibition unraveled by present QSAR evaluation are interconnected and thus easy to incorporate to optimize present LSD1 inhibitors towards more potent analogues; for example, a higher number of Nitrogen atoms precisely at six bonds and a lower number of Hydrogen atoms at three bonds from the ring Carbon atom can be introduced at the same time to optimize the LSD1 inhibitors towards better activity, and a higher number of non-ring Oxygen atoms precisely at nine bonds from the amide Nitrogen and a less frequent occurrence of sp2 Oxygen within 4Å boosts the LSD1 inhibitory activity. Likewise, the hydrogen bond donor atom at two bonds and amide Nitrogen at four bonds from sp3 hybridized Carbon atoms enhances the desired activity. Two of the five descriptors in the split-set model emphasize the relevance of the ring carbon atom, whereas one descriptor represents the importance of the ring Sulphur atom, indicating that there is room for modification of dataset compounds for greater LSD1 inhibition. On the other hand, two out of five descriptors emphasize the relevance of amide nitrogen, suggesting that the current dataset compounds might be optimized for improved LSD1 inhibition. Lipophilic atoms, such as ring carbon atoms, were identified as a possible center for the optimization of LSD1 inhibitors for anticancer efficacy by certain chemical descriptors. As a result, the created QSAR model may be used to improve compounds for better LSD1 inhibition and cancer prevention. The docking results revealed that the descriptors, **fdonsp3C2B** and **lipo_ringS_8Bc,** played important roles in the inhibition of the LSD1 receptor, which was consistent with the QSAR findings. The MD simulation results display that the ligands were tightly bound to the binding site of the receptor during the simulation. The ligands are still firmly connected to the receptor’s binding site, as evidenced by the fact that the RMSD values for the ligand fit-to-protein does not significantly vary over the course of the simulation. Compound **1**’s position was altered in the last 150-ns frame of the MMGBSA simulation, compared to the 0-ns trajectory, indicating a more advantageous binding pose for the binding cavity of the protein. Therefore, the MD simulation and MMGBSA analysis strengthens the outcome of the QSAR and Molecular docking studies.

## Figures and Tables

**Table 1:** Binding energy calculation of compound **1** with LSD and non-bonded interaction energies from MMGBSA trajectories. (star indicates mean of all th energy value).

Energies (kcal/mol) Mean *	LSD1 + Comp-1
ΔG_bind_	−42.18 ± 7.60
ΔG_bind_Lipo	−07.62 ± 4.78
ΔG_bind_vdW	−15.63 ± 7.77
ΔG_bind_Coulomb	−12.54 ± 4.07
ΔG_bind_H_bond_	−11.68 ± 2.00
ΔG_bind_SolvGB	31.53 ± 9.70
ΔG_bind_Covalent	10.22 ± 4.00

## Data Availability

The data is available in the Supplementary section.

## Supplementary Materials

The following supporting information can be downloaded at: https://www.mdpi.com/article/10.3390/molecules27154758/s1. Table S1: The SMILES notation for 84 leads, along with their reported **EC_50_** and **pEC_50_** values. Table S2: The values for selected molecular descriptors present in QSAR models.

## Abbreviations

CADD	Computer Aided Drug Designing
SMILES	Simplified Molecular-Input Line-Entry System
GA	Genetic Algorithm
MLR	Multiple Linear Regression
QSAR	Quantitative Structure-Activity Relationship
QSARINS	QSAR Insubria
OECD	Organization for Economic Co-operation and Development
OFS	Objective Feature Selection
SFS	Subjective Feature Selection
LSD1	Lysine-specific demethylase 1

## References

[1] Maiques-Diaz A., Somervaille T.C.P. (2016). LSD1: Biologic roles and therapeutic targeting. Epigenomics.

[2] McGrath J.P., Williamson K.E., Balasubramanian S., Odate S., Arora S., Hatton C., Edwards T.M., O’Brien T., Magnuson S., Stokoe D. (2016). Pharmacological Inhibition of the Histone Lysine Demethylase KDM1A Suppresses the Growth of Multiple Acute Myeloid Leukemia Subtypes. Cancer Res..

[3] Mohammad Helai P., Smitheman Kimberly N., Kamat Chandrashekhar D., Soong D., Federowicz Kelly E., Van Aller Glenn S., Schneck Jess L., Carson Jeffrey D., Liu Y., Butticello M. (2015). A DNA Hypomethylation Signature Predicts Antitumor Activity of LSD1 Inhibitors in SCLC. Cancer Cell.

[4] Ma L.-Y., Zheng Y.-C., Wang S.-Q., Wang B., Wang Z.-R., Pang L.-P., Zhang M., Wang J.-W., Ding L., Li J. (2015). Design, Synthesis, and Structure–Activity Relationship of Novel LSD1 Inhibitors Based on Pyrimidine–Thiourea Hybrids As Potent, Orally Active Antitumor Agents. J. Med. Chem..

[5] Cherkasov A., Muratov E.N., Fourches D., Varnek A., Baskin I.I., Cronin M., Dearden J., Gramatica P., Martin Y.C., Todeschini R. (2014). QSAR Modeling: Where Have You Been? Where Are You Going To?. J. Med. Chem..

[6] Fujita T., Winkler D.A. (2016). Understanding the Roles of the “Two QSARs”. J. Chem. Inf. Model..

[7] Abdizadeh R., Heidarian E., Hadizadeh F., Abdizadeh T. (2021). QSAR Modeling, Molecular Docking and Molecular Dynamics Simulations Studies of Lysine-Specific Demethylase 1 (LSD1) Inhibitors as Anticancer Agents. Anti-Cancer Agents Med. Chem..

[8] Fang Y., Liao G., Yu B. (2019). LSD1/KDM1A inhibitors in clinical trials: Advances and prospects. J. Hematol. Oncol..

[9] Gaulton A., Hersey A., Nowotka M., Bento A.P., Chambers J., Mendez D., Mutowo P., Atkinson F., Bellis L.J., Cibrián-Uhalte E. (2017). The ChEMBL database in 2017. Nucleic Acids Res..

[10] Vianello P., Sartori L., Amigoni F., Cappa A., Fagá G., Fattori R., Legnaghi E., Ciossani G., Mattevi A., Meroni G. (2017). Thieno[3,2-b]pyrrole-5-carboxamides as New Reversible Inhibitors of Histone Lysine Demethylase KDM1A/LSD1. Part 2: Structure-Based Drug Design and Structure–Activity Relationship. J. Med. Chem..

[11] Zheng Y.-C., Ma J., Wang Z., Li J., Jiang B., Zhou W., Shi X., Wang X., Zhao W., Liu H.-M. (2015). A Systematic Review of Histone Lysine-Specific Demethylase 1 and Its Inhibitors. Med. Res. Rev..

[12] Niwa H., Sato S., Hashimoto T., Matsuno K., Umehara T. (2018). Crystal Structure of LSD1 in Complex with 4-[5-(Piperidin-4-ylmethoxy)-2-(p-tolyl)pyridin-3-yl]benzonitrile. Molecules.

[13] Stazi G., Zwergel C., Valente S., Mai A. (2016). LSD1 inhibitors: A patent review (2010–2015). Expert Opin. Ther. Pat..

[14] Magliulo D., Bernardi R., Messina S. (2018). Lysine-Specific Demethylase 1A as a Promising Target in Acute Myeloid Leukemia. Front. Oncol..

[15] O’Boyle N.M., Banck M., James C.A., Morley C., Vandermeersch T., Hutchison G.R. (2011). Open Babel: An open chemical toolbox. J. Cheminform..

[16] Masand V.H., Rastija V. (2017). PyDescriptor: A new PyMOL plugin for calculating thousands of easily understandable molecular descriptors. Chemom. Intell. Lab. Syst..

[17] Gramatica P., Chirico N., Papa E., Cassani S., Kovarich S. (2013). QSARINS: A new software for the development, analysis, and validation of QSAR MLR models. J. Comput. Chem..

[18] Masand V.H., Mahajan D.T., Nazeruddin G.M., Hadda T.B., Rastija V., Alfeefy A.M. (2014). Effect of information leakage and method of splitting (rational and random) on external predictive ability and behavior of different statistical parameters of QSAR model. Med. Chem. Res..

[19] Consonni V., Todeschini R., Ballabio D., Grisoni F. (2019). On the Misleading Use of QF32 for QSAR Model Comparison. Mol. Inform..

[20] Krstajic D., Buturovic L.J., Leahy D.E., Thomas S. (2014). Cross-validation pitfalls when selecting and assessing regression and classification models. J. Cheminform..

[21] Martin T.M., Harten P., Young D.M., Muratov E.N., Golbraikh A., Zhu H., Tropsha A. (2012). Does Rational Selection of Training and Test Sets Improve the Outcome of QSAR Modeling?. J. Chem. Inf. Model..

[22] Chirico N., Gramatica P. (2012). Real External Predictivity of QSAR Models. Part 2. New Intercomparable Thresholds for Different Validation Criteria and the Need for Scatter Plot Inspection. J. Chem. Inf. Model..

[23] Roy P.P., Kovarich S., Gramatica P. (2011). QSAR model reproducibility and applicability: A case study of rate constants of hydroxyl radical reaction models applied to polybrominated diphenyl ethers and (benzo-)triazoles. J. Comput. Chem..

[24] Mimasu S., Sengoku T., Fukuzawa S., Umehara T., Yokoyama S. (2008). Crystal structure of histone demethylase LSD1 and tranylcypromine at 2.25Å. Biochem. Biophys. Res. Commun..

[25] Hollingsworth S.A., Karplus P.A. (2010). A fresh look at the Ramachandran plot and the occurrence of standard structures in proteins. BioMol. Concepts.

[26] Gaudreault F., Morency L.-P., Najmanovich R.J. (2015). NRGsuite: A PyMOL plugin to perform docking simulations in real time using FlexAID. Bioinformatics.

[27] Gaudreault F., Najmanovich R.J. (2015). FlexAID: Revisiting Docking on Non-Native-Complex Structures. J. Chem. Inf. Model..

[28] Bowers K.J.A.C., David E., Xu H., Dror Ron O., Eastwood Michael P., Gregersen Brent A., Klepeis John L., Kolossvary I., Moraes M.A., Sacerdoti Federico D. Scalable Algorithms for Molecular Dynamics Simulations on Commodity Clusters. Proceedings of the 2006 ACM/IEEE Conference on Supercomputing.

[29] Ferreira L.G., Dos Santos R.N., Oliva G., Andricopulo A.D. (2015). Molecular docking and structure-based drug design strategies. Molecules.

[30] Hildebrand P.W., Rose A.S., Tiemann J.K.S. (2019). Bringing Molecular Dynamics Simulation Data into View. Trends Biochem. Sci..

[31] Rasheed M.A., Iqbal M.N., Saddick S., Ali I., Khan F.S., Kanwal S., Ahmed D., Ibrahim M., Afzal U., Awais M. (2021). Identification of Lead Compounds against Scm (fms10) in Enterococcus faecium Using Computer Aided Drug Designing. Life.

[32] Shivakumar D., Williams J., Wu Y., Damm W., Shelley J., Sherman W. (2010). Prediction of Absolute Solvation Free Energies using Molecular Dynamics Free Energy Perturbation and the OPLS Force Field. J. Chem. Theory Comput..

